# Performance of Graphene-Based and Polyether-Ether-Ketone Polymers as Removable Partial Denture Esthetic Clasp Materials after Cyclic Fatigue

**DOI:** 10.3390/polym14152987

**Published:** 2022-07-23

**Authors:** Mostafa Omran Hussein

**Affiliations:** Department of Prosthodontic Sciences, College of Dentistry in Ar Rass, Qassim University, El-Qassim 58876, Saudi Arabia; m.omran@qu.edu.sa; Tel.: +96-655-727-9282

**Keywords:** PEEK, graphene oxide, partial denture, fatigue, nanotechnology, clasp, esthetic material

## Abstract

The esthetic clasp material is a clinical demand for a satisfactory removable partial denture. The purpose of this study is to assess the mechanical performance of graphene-based polymer (GBP) and polyether-ether-ketone (PEEK) materials as clasp materials. Thirty-two clasps were fabricated by CAD-CAM from two materials, GBP and PEEK. All clasps were tested for retention force after 10,000 cycles of insertion and removal and thermocycling. The clasp arms’ deformation was measured, and areas of stress–strain concentration were explored. The Mann–Whitney U test was used to compare the retentive force of the studied groups, while the independent sample *t*-test was applied to check the difference in clasp arm deformation at α = 0.5. The results showed a significantly higher retentive force (2.248 ± 0.315 N) in PEEK clasps, at *p* < 0.001. The deformation of the clasp arm of the GBP clasps was significantly higher than PEEK clasps. Areas of stress–strain concentration were seen at the junction of the retentive arm to the minor connector and at the retentive arm terminal. It could be concluded that PEEK polymer had a better mechanical performance as an esthetic clasp material than the GBP. An optimization study for GBP might be required to check the validity of such an application.

## 1. Introduction

The removable partial denture (RPD) has been used as a successful treatment for many decades. It is indicated in several clinical conditions that tooth-supported or implant-supported fixed partial dentures might not be suitable [1,2]. The removable partial denture is the preferred treatment option for patients having few remaining teeth, poor periodontal condition, inadequate residual bone for implant placement, and those who underwent bone grafting as a temporary prosthesis. In addition, medical, psychological, and financial conditions may also affect the treatment decision [2,3,4].

Although the basic principles of the RPD design are the same, developments in the framework material are under study [5,6,7,8,9,10]. Studies are focused on using new materials that could resolve the shortcomings of the alloys currently used in framework fabrication. The major shortcomings of the alloys such as cobalt-chromium (Co-Cr) are the unesthetic color of the material, corrosion tendency, and harmful effect on the periodontal ligaments because of high rigidity [11]. Recently, many esthetic materials, such as acetal resin, aryl ketone, ceria-stabilized zirconia/alumina nanocomposite, polyester, polyamide, polyaryl-ether-ketone, and polyether-ether-ketone have been used to replace the framework alloys [1,6,8,11,12,13,14,15]. These esthetic materials were not only able to exchange the metal display of the RPD alloys but also minimize the forces on the abutment teeth because of their flexibility [5,10]. As they are polymeric materials, there is no metallic taste because of corrosion and no chance for galvanism with other alloys in the oral cavity [10,16]. 

Polyether-ether-ketone (PEEK) material is a polymer used in several dental applications, such as fixed, removable, and maxillofacial prosthodontics. Its applications are also extended to implant dentistry, endodontics, and orthodontics [5]. This polymer showed favorable characteristics that are suitable for many dental applications. For example, it has an elastic modulus equal to 4100 MPa at normal body temperature (37 °C), which could be increased by modifiers such as carbon fibers and zirconia nanocomposite [10,17,18]. It also has a high flexural strength ranging from 110 to 170 MPa but lower hardness than alloys and ceramics [5]. In addition, it showed good biocompatibility and radiolucency, which minimizes tissue reaction and reduces artifacts during imaging [5,10]. It has two physical forms that have been used frequently in dentistry, the milled and the injectable forms. The first one comes as different colored computer-aided design–computer-aided machine (CAD-CAM) discs and the second as granules or in pellet form [16,19,20].

Graphene nanomaterial is a new material generated by nanotechnology for materials’ enhancement. There are two known compounds produced from graphene, graphene oxide (GO) and its reduced form (GOr) [9,21,22,23,24,25,26]. Both compounds can combine with different polymers and provide several enhancement forms [9,24]. For example, GO was successful in improving tensile strength, elastic modulus, and both fracture toughness and energy of polymers such as polycarbonate and epoxy resin [9]. It was also beneficial to improve the mechanical properties of the polymethyl methacrylate (PMMA) [24]. Moreover, it was effective as an antimicrobial substance in dental applications [23,27]. Therefore, it has been used in several applications, such as prosthodontics, orthodontics, restorative dentistry, and implant dentistry [21,22,24,25,26]. After producing the graphene-based polymer in milled form, the material became more popular in dentistry. This material is a type of graphene-doped PMMA manufactured in dentistry as discs to be milled by the CAD-CAM procedure from previously 3D-designed parts [28].

Many studies evaluated different materials used as RPD framework, especially the direct retainer component [4,7,11,12,15,19,20,29,30,31,32]. Most of the methodologies have applied cantilever bending, three-point loading, cyclic fatigue of insertion and removal of the actual clasp, and virtual stress–strain analysis [13,17,33,34,35]. Testing was performed after thermocyclic aging in distilled water or artificial saliva ranged between 5000 to 15,000 cycles. The retentive pull-off forces were recorded at several operator-specified cycles, with a total of 5000 to 20,000 cycles [11,19,20]. The analog tooth was fabricated from a cast alloy, extracted tooth, or lithium disilicate crowns. Clasp deformation due to loading was also investigated by micro-computerized tomography (µ CT), statistically deformable models, and calculation of the total deformation by stress analysis software [11,13,17,29,33]. Scanning electron microscopy was also a beneficial method for studying the surface changes because of friction on both the clasp fitting surface and the counter analog tooth surface [7,11,29,31].

To date, no study has evaluated the validity of the graphene-based polymer (GBP) as an esthetic RPD clasp material and compared the results with the PEEK material in a similar condition. Therefore, the aim of the current study was to assess the mechanical performance of the GBP and PEEK materials used as RPD clasp materials by testing their cyclic pull-off force after aging. In addition, the amount of clasp arms’ deformation was measured, and areas of stress and strain were explored. The tested null hypothesis was that there would be no difference in the pull-off force, clasp arm deformation, and areas of stress and strain between PEEK or GBP polymers when used as RPD clasp materials.

## 2. Materials and Methods

Assessment of the tested materials (PEEK and GBP) was designed in three phases. Testing the retention force of the clasps after cyclic insertion and removal, measuring the clasp arms’ deformation, and finally, estimating areas of high stress and strain using stress–strain analysis. The material used for fabrication of the premolar analogue and its base was chromium-cobalt alloy (Wironium Super hard, Bego, Bremen, Germany), while the tested clasp materials were PEEK (Disco Smile PEEK, Disco, Italy) and graphene-based polymer (G-CAM, Graphenano Dental S.L., Valencia, Spain). Material properties of the polymers used are presented in Table 1. 

### 2.1. Design and Manufacturing of the Samples

The three-dimensional model of a premolar tooth 15# was modified by mesh-editing software (Meshmixer v 3.2.37; Autodesk, San Rafael, CA, USA) to have a cuboid base with dimensions of 11.5 × 9.5 × 12.3 mm. This base served as a model gripping during testing. In addition, the distal surface of the premolar, the occluso-distal part, as well as the retentive buccal undercut were virtually prepared by sculpting tools to receive a guiding plate, distal occlusal rest, and retentive terminal at a 0.75 mm undercut, respectively. The 3D model was milled from wax by a CAM machine (DWX-52D, Roland DGA, Irvine, CA, USA) followed by casting from chromium-cobalt alloy (Wironium Super hard, Bego, Bremen, Germany) by the lost wax technique. The metallic model was then sprayed with anti-glare material (EZ Scan; Alphadent, Waregem, Belgium) with a 3 µm particle size powder before 3D scanning. Using a desktop 3D scanner (E4, 3Shape, Copenhagen, Denmark), the model was scanned and exported as a Standard Tessellation Language file (STL). 

**Figure 1:**
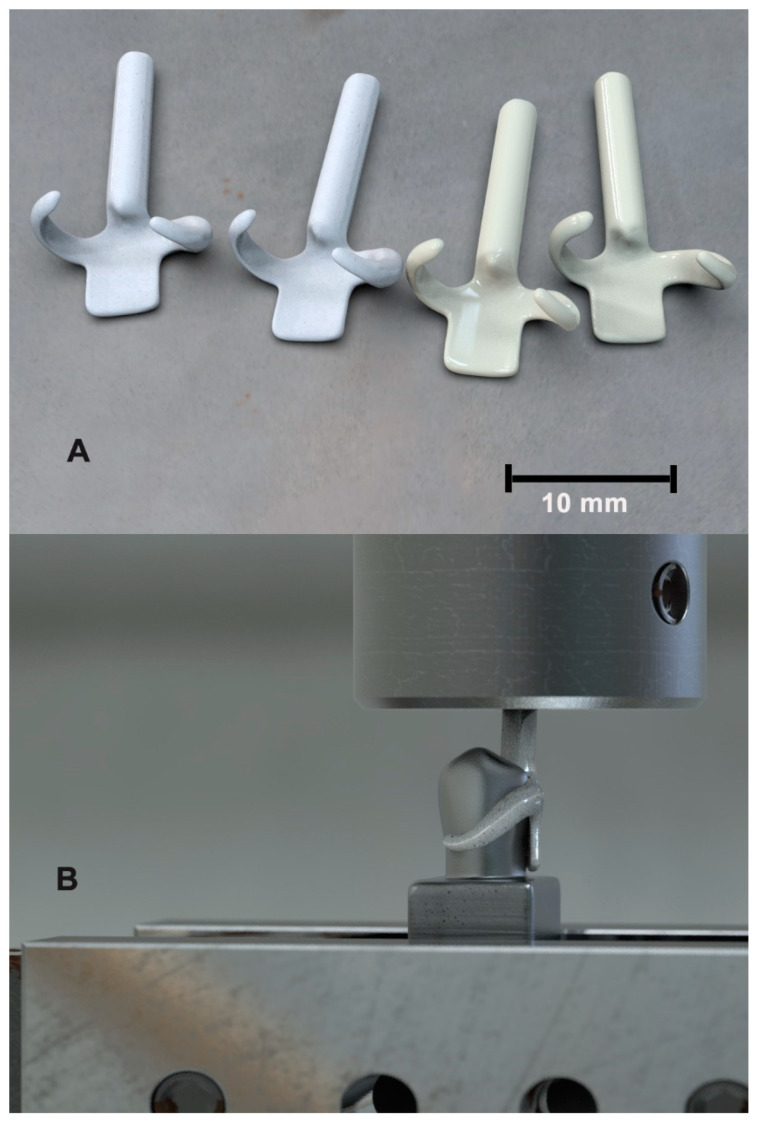
(**A**) Retentive and reciprocal arms of the CAD-CAM-prepared clasps with their attaching rods. (**B**) The manufactured clasp and tooth assembly attached in place in the testing machine.

A simple Aker clasp was designed on the premolar 3D model by dental CAD design software (DentalCAD; Exocad Matera, Exocad GmbH), including a retentive arm (6 × 2.3 × 1.7 mm), a reciprocal arm (5.5 × 1.8 × 1.7 mm), a guiding plate (4.5 × 4.9 × 1 mm), and distal occlusal rest (2.0 × 4.8 mm) [19,20]. A vertical cylinder was modeled at the insertion axis of the clasp and merged on the designed rest to grip the clasp during testing. The specimens were milled from the designed model (N = 32, 16 per group) by the CAM machine from the two studied disc materials, PEEK (Disco Smile PEEK, Disco, Italy) and graphene-based polymer (G-CAM, Graphenano Dental S.L., Valencia, Spain) (Figure 1a). 

The clasp samples were subjected to storage and aging in an incubator (Fisher Scientific, Waltham, MA, USA) in the distilled water at 37 °C for 30 days. The aging process was performed under cycles (10,000 cycles) of fluctuant temperature changes with a dwell time of 20 s and temperature ranging from 5 to 55 °C using a thermocycling machine (Thermocycling K178, Tokyogiken, Tokyo, Japan) [19,39].

### 2.2. Testing the Retention Force

The retention force test was conducted by adjusting the premolar models with the insertion direction of the clasp in the machine adaptor of the universal testing machine (Zwick 1445, Zwick Co., Ulm, Germany) (Figure 1b). The clasp sample was attached to the counterpart of the machine to meet the premolar model in a pull-off direction. The clasp was tested for a passive set on the analog tooth by the device sensor, showing a zero Newton value on the display. The pull-off process was repeated in cycles, where the recording of the retentive force followed a pre-customized sequence throughout the 10,000 cycles. The retentive force’s recordings were scheduled in the load cell to be for the first 10 cycles, after every 10 cycles for the next 90 cycles, and after every 100 cycles for the remaining cycles [11]. All readings were collected and plotted as a retentive force against the log of the number of cycles applied.

### 2.3. Measuring the Clasp Arms’ Deformation

This study was conducted by monitoring the 3D deviation of the clasps’ arms from their initial shape (pre-loading) to the final deviation (post-loading), occurring especially at the terminal part. To fulfill this process, the clasp samples were 3D-scanned before and after mechanical testing using a 3D scanner (E4, 3Shape, Copenhagen, Denmark). An accurate metrology software (Geomagic Control X v 2018.1.1; 3D Systems, Rock Hill, SC, USA) was used to align each clasp model before and after mechanical testing to their originally designed 3D model (reference entity) based on the best fit alignment of the undeformed part. Eight equally separated fixed points at the inner surface of the terminal part of each clasp arm (retentive and reciprocal) were assigned as comparison points. The gap distance between points at the pre-loaded clasp model and their corresponding post-loaded clasp was measured automatically. All gap distance values for both materials at the retentive and reciprocal arms were collected and statistically analyzed. 

### 2.4. Exploring Areas of High Stress and Strain Using Finite Element Analysis

To focus on areas of higher stress and strain of the clasp arms, a finite element simulation of the pull-off process was conducted. The previously designed 3D models of the premolar tooth and its overlying clasp were imported into the finite element software (ANSYS workbench v 21, ANSYS). The models have been meshed, forming 126,093 nodes and 72,562 elements based on the convergence study. The average retentive force recorded from the mechanical study for each material was applied, while the model of the premolar was fully constrained during the analysis. The inner surfaces of the clasp could slide on the tooth surfaces with a frictional contact at a 0.3 frictional coefficient [18]. The engineering material library was changed by adding three new materials corresponding to the studied scenario (Table 1) [40]. The Young’s modulus and Poisson’s ratio of the chromium-cobalt alloy were 220 GPa and 0.3, respectively. All materials are assumed to have an isotropic behavior. The maximum equivalent stress and strain of both clasp materials were assigned as post-processing outcome values. 

### 2.5. Statistical Analysis

A total sample size of 32 clasps (16 for each group) was selected at an effect size = 1.2 and an actual power = 95% (α err prob. = 0.05) using sample power analysis software (G*Power v3.1.9.4 software; Heinrich-Hein-University, Dusseldorf, Germany). The frequency of retentive force data showed non-parametric distribution by the Shapiro–Wilk test. Accordingly, the Mann–Whitney U test was used to compare the studied groups at α = 0.5. The data of the clasp arms’ deformations showed normal distribution. The independent sample *t*-test was applied to check the difference between the means of retentive arms of the studied materials as well as their reciprocal arms at α = 0.5.

## 3. Results and Discussion

The aim of the current study was to evaluate the difference in the retentive force and the deformation after cyclic aging and pull-off force of the RPD clasps manufactured from PEEK and GBP materials, followed by exploring the areas of maximum stress and strain. 

The current study was conducted to assess the use of GBP material as a clasp material after the success of the PEEK material to be an esthetic replacement to Co-Cr material in such application [13,19,20,29]. Recently, graphene-based polymer was introduced as milled CAD-CAM discs that are appropriate for several dental applications [28]. It had the sufficient strength to be used as tooth-supported and implant-supported fixed and fixed-detachable prostheses. It demonstrated favorable mechanical properties that suit restorative and orthodontic applications [21,22,24,25,26]. In addition, the material had antimicrobial activity, which could be the key element in enhancing the biological environment and health of the surrounding tissue [23,27]. This property is a great contribution compared to other polymers used in similar applications, such as PEEK. In the current study, GBP was tested as an esthetic clasp material, which, if successful, will have a good chance of minimizing plaque accumulation, clinically. 

A maxillary first premolar tooth was selected because of its location, which mostly has an esthetic consideration [11,32]. The analog tooth material selected was cast Cr-Co material due to its rigidity and hardness that facilitate standardization of the testing process [19,20,32]. To represent frequent RPD insertion and removal by the patient and simulate the effect of the oral environment, cyclic pull-off forces for 10,000 cycles and aging for 10,000 thermocycles were applied [19,20]. Furthermore, the retentive force recording steps were distributed throughout the loading cycles as it was believed that these steps could represent the expected changes occurring in the retentive force [11]. 

### 3.1. Results of the Retention Force

**Figure 2:**
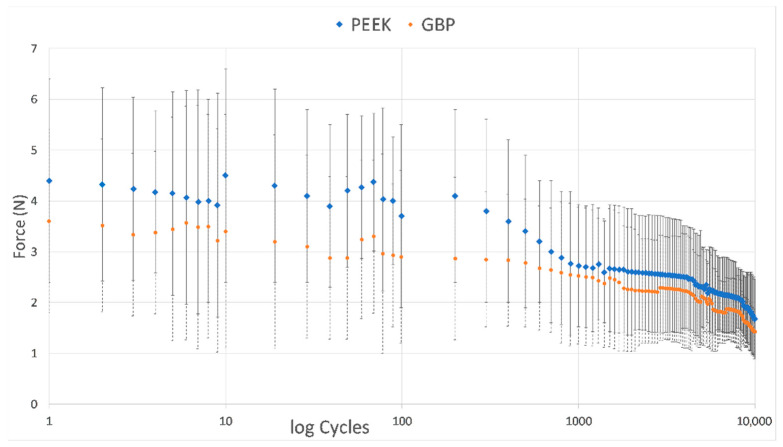
Plot showing the mean and standard deviation of the pull-off force in Newtons (N) of the studied materials at the selected steps of removal cycles. The blue diamond symbol with a continuous line represents the PEEK clasps and the orange circle symbol with a dotted line represents the GBP clasps.

The retentive force data in Newtons (N) recorded at the pre-determined cyclic steps were collected and plotted. The chart revealed more retentive forces throughout all recorded steps in the PEEK material clasps than seen in the GBP clasps (Figure 2). The retentive force of the PEEK clasp material showed a statistically significantly higher value (2.248 ± 0.315 N) than the GBP clasp material (2.018 ± 0.298 N), at *p* < 0.001 (Table 2, Figure 2). 

The results of the retentive force cycles showed a gradual decrease in the retentive force for both materials, with few outlier values (Figure 2). Especially after 10, 70, and 200 cycles, the retentive force seems to increase, breaking the gradual decrease seen before. This finding was also seen in a previous study, where an increase was recorded after the first phase of the pull-off cycles of a total of 15,000 cycles [11,32]. The authors recorded the same finding even at different thicknesses. Some authors claimed a change in the clasp retentive force to the adaptation process of the clasp after a short time of RPD usage, where scratches build on the fitting surface of the clasp, increasing friction and leading to more retentive force that declines soon after wear. They clarified that this effect explained the need for clasp adjustment in the follow-up visits after RPD insertion [7,11,41]. This gradual decrease in the retentive force might also be due to the viscoelastic nature of the tested polymers. This behavior matched the stress-relaxation that happened during loading of the polymeric materials.

In the current study, the ranges of the retentive force of PEEK and GBP materials were 3.70–1.68 and 2.90–1.43 N, respectively (Table 2). These values were close to the clasp retentive force of polymeric materials tested in some studies, especially for pressed and milled PEEK materials, and slightly lower than other literature values [6,8,11,12,15,19,20,29,30,31,34]. As there is no standardized method for clasp retention measurement, variations could be seen between different studies, and therefore the comparison should be limited to articles with the same design, such as analog material, aging method, rate of displacement, number of pull-off cycles, clasp attachment site, amount of undercut tested, and shape of the specimens [7,11,12,29,32,41]. Although, the important value that should be recognized is the value of the minimum retentive force that is acceptable for clinical use. Whereas some authors believed that 2.3 N was enough for clasp retention [29], others recommend keeping values between 3 and 5 N to avoid periodontal ligament trauma that occurs at 10 N [4]. Peng et al. [13], in a more in-depth study about PEEK clasp optimization, confirmed that 1.6 N is the minimum value required clinically for clasp retention. This finding coincides with the minimum values of the current study recorded for PEEK material (1.68 N). However, this value is higher than the minimum value recorded for GBP material (1.43 N). Accordingly, the use of the GBP in the current dimensions and undercut depth is not satisfactory and needs to be optimized for successful clinical use, as performed before for PEEK material [13]. Adding graphene material improved the resin elastic modulus from 2850 to 3200 MPa and reduced its high residual strain, and therefore it became less vulnerable to fracture [12]. However, it is less than the PEEK elastic modulus (4100 MPa) [17,24,26]. This could be seen clearly in Figure 2, where the graph shows a higher level for PEEK material than GBP in all measured cycles [34]. It was believed that the thickness, width, length, and depth of the undercut could play a major role in the stress generated [1,13]. The optimization process may encompass increasing the clasp dimensions and may limit its use to certain clinical conditions [13,15,35].

### 3.2. Results of the Clasp Arms’ Deformation Measurements

**Figure 3:**
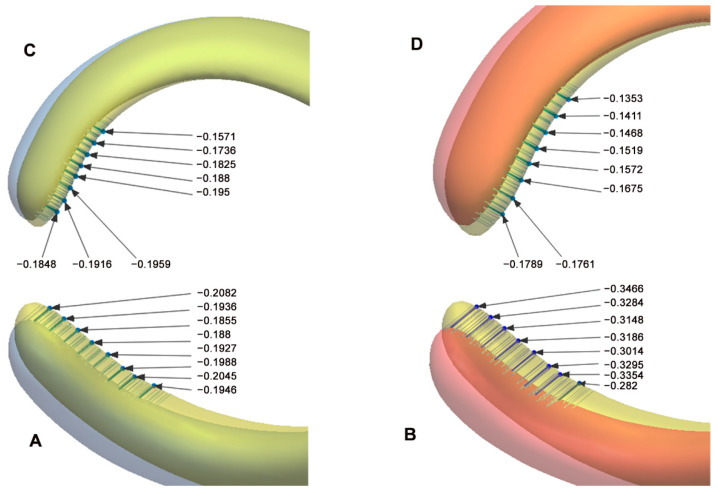
(**A**,**B**) Deviation analysis of the retentive arms of the PEEK and GBP materials, respectively. (**C**,**D**) deviation analysis of the reciprocal arms in the same sequence.

The mean and standard deviation of the deformation in the retentive arm of the GBP clasp material showed a statistically higher value (0.220 ± 0.069 mm) than the PEEK clasp material (0.158 ± 0.047 mm), at *p* = 0.007 (Table 3 and Figure 3A,B). Similarly, the mean and standard deviation of the deformation recorded at the GBP reciprocal arms showed a higher value (0.187 ± 0.0376 mm) than the PEEK clasp material (0.133 ± 0.034 mm), with a significant value of *p* < 0.001 (Table 3 and Figure 3C,D). The retentive arms’ deformation was higher than those of the reciprocal arms, with more deformation present in the GBP clasp arms.

Studying the clasp arms’ deformation revealed a significant difference between the studied materials. Graphene-based polymer showed higher deformation than PEEK material, with higher values in retentive arms than reciprocal arms in both materials (Figure 3, Table 3). This finding was expected and could be attributed to the difference in the rigidity between the studied materials. The clasps were tested for 10,000 cycles, which were estimated to equal 6.5 years of oral function [29,30,31,34]. The circumferential clasp selected for the current study is one of the simplest clasps used in RPD. It has two arms: The retentive arm, which extends from the minor connector above the survey line and once it has enough taper, it crosses the survey line to the undercut area on the opposite side. It keeps passive until insertion and removal. During insertion and removal of the clasp, the retentive arm exerts flexion to allow exit from the undercut, while the second arm, the reciprocal arm, just reciprocates the effect of the retentive arm on the tooth while keeping the arm above the undercut area. The reciprocal arm also has more rigidity than the retentive arm, as it has a less taper and length. Therefore, the retentive arm exerts more displacement than the reciprocal arm and is more flexible, so it is expected to have more deformation [1,33,41]. By the end of 10,000 cycles, the clasp arms showed deformation at the clasp tips, expressing the arms’ fatigue (Figure 3). This finding was in harmony with Marie et al.’s [11] study, where the retentive and reciprocal arms of both metallic and polymeric materials showed different levels of deformation for both axial and off-axial pull-off forces. Despite the difference in the materials tested and the direction of pull-off forces, it should be noted that the range of values in the current study was within the range of their study. It should also be mentioned that they selected a small, localized area at the tip of the arms, while the current study located eight separate points on the arms’ terminals (Figure 3). The current study also matched the finding in another study about the higher deformation seen in the retentive than the reciprocal arm [4].

### 3.3. Location of High Stress and Strain Areas

**Figure 4:**
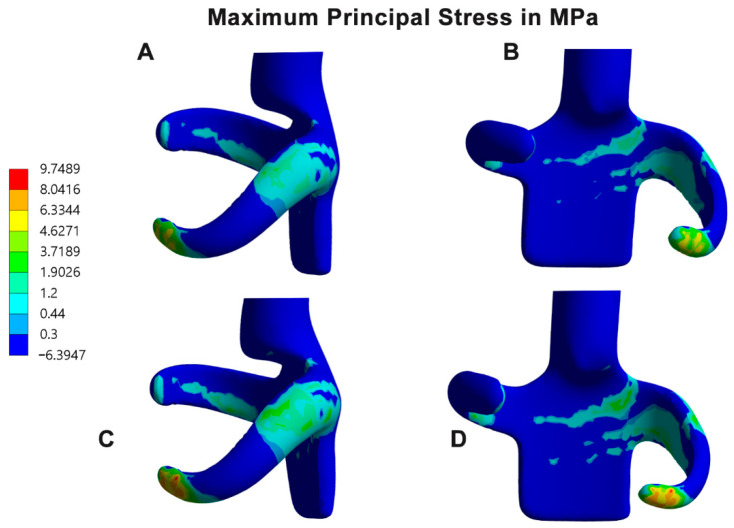
(**A**,**B**) Scaled color map of the maximum principal stress (in MPa) of the PEEK clasp showing areas of higher stress from buccal and mesial views, respectively. (**C**,**D**) Corresponding views of the GBP clasp.

**Figure 5:**
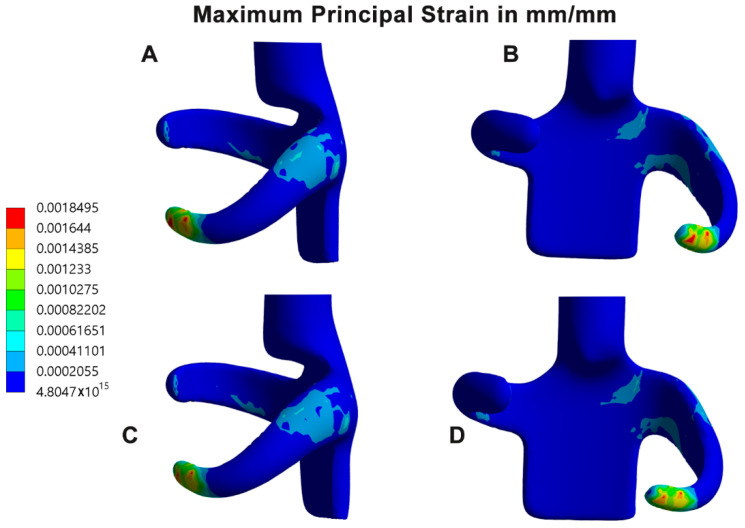
(**A**,**B**) Scaled color map of the maximum principal strain (in mm/mm) of the PEEK clasp showing areas of higher stress from buccal and mesial views, respectively. (**C**,**D**) Corresponding views of the GBP clasp.

Finite element analysis of the clasp materials revealed higher maximum principal stress (12.66 MPa) in the GBP clasp than in the PEEK clasp, especially at the retentive terminals where stress concentration bands are clearly recognized. In addition, some bands of stress concentrations were also seen at the junction between the guiding plate minor connector and the retentive arm, with few extensions toward the retentive arm. Fewer bands could also be recorded at the tissue side of the reciprocal arms (Table 4, Figure 4A–C). There were some similarities between the color bands of the PEEK clasp material and the GBP material, but with more intense color bands and values in the GBP clasp. In contrast, the maximum principal strain of the GBP (3.331 × 10^−3^ mm/mm) showed higher strain bands than the PEEK material (1.85 × 10^−3^ mm/mm), specifically at the terminal part of the retentive arm and the connection of the retentive arm and the guiding plate (Table 4, Figure 5A–C).

Exploring the areas of stress and strain in the clasps showed two major areas of interest (Figure 4 and Figure 5). They were clearly seen surrounding the junction between the guiding plate minor connector and the retentive clasp arm and at the retentive terminal of the retentive arm. As mentioned before in this discussion, during clasp insertion and removal, the retentive arm flexes to get in and out of the undercut, leading to stress and strain concentrations in the mentioned junction. In the same context, the retentive terminal and the reciprocal arm are subjected to friction, which is magnified as the retentive terminal crosses the survey line to the undercut areas. These findings coincide with previous studies showing bands of stress concentration at the junction of the clasp arm with the minor connector [1,13,17,29,33]. It is also in harmony with the deformation pattern seen mostly in the arm terminals as a response to the arm fatigue [11,33]. In contrast, another study reported an increase in stress at the middle of the arm rather than the terminals [7]. Although, their study was conducted on one simulated arm attached to a fixed connector, which may not represent the clinical condition. In a 10-years clinical study, Wagner et al. [2] evaluated the RPD line of treatment. They confirmed that one of the main causes of failure was clasp fatigue and fracture at the junction of the retentive arm and the minor connector in circumferential clasps of RPD included in their study.

Based on this study’s result, the difference in the recorded retentive force after 10,000 cycles was significantly higher in the PEEK clasps than in the GBP clasps. In addition, the values of the deformation that happened in the retentive and reciprocal arms in the PEEK clasps were also significantly higher than in the GBP clasp arms. Therefore, the null hypothesis of the previously tested data was rejected. 

The current study had some limitations, such as the type of material used for the analog tooth. Other studies used the natural teeth or the lithium disilicate. Their studies relied on using more natural clinical scenarios, but the current study preferred to keep standardization and avoid wear that could happen on the analog tooth surface [11,30,31]. It will be more valuable to study the effect of using different restorative materials at the analogue tooth on the GBP clasps within a full optimization study in the future [13,30]. The current study also focused on assessment of the GBP versus the PEEK material only in axial insertion and removal. Therefore, testing the material in different insertion and removal directions to mimic the fault use of the patients will be beneficial [11]. It should also be mentioned that this in vitro study did not consider the function of the periodontal ligament and their damping action during function. The influence of stresses during mastication, as well as representing the role of PDL, was also neglected, and could be considered in the future studies. In-depth study of the clasp fitting surface using scanning electron microscopy could also enrich the future studies to clarify the pattern of wear occurring during cyclic insertion and removal [11]. Clinical evaluation of the GBP after the optimization study should be considered, checking its validity in long-term clinical situations. 

## 4. Conclusions

Graphene-based polymer was recently applied in dentistry and has demonstrated favorable outcomes. The current study was conducted to assess the use of the graphene-based polymer as an esthetic RPD clasp material and compare its results with PEEK polymer, which has been successfully used in such applications. Therefore, within the limitations of this in vitro study, the following conclusion could be drawn:

Polyether-ether-ketone showed better retentive force and less deformation than graphene-based polymer after cyclic fatigue, simulating RPD clasp insertion and removal. Areas of stress and strain concentrations were similar for both materials, which were the frequent areas of clasp failure. More efforts are required to optimize the graphene-based polymer before using it clinically as a valid esthetic clasp material. 

## Figures and Tables

**Table 1:** Material properties of the tested polymers [13,36,37,38].

Material	Young’s Modulus (MPa)	Poisson Ratio	Bending Strength (MPa)	Surface Hardness (Shore)	Water Sorption (µg/mm^3^)	Glass Transition Temp. (Tg) °C
GBP	3200	0.3	140	88	4	120
PEEK	4100	0.4	183	87.2	1.7	143 *

* Tg measured by DSC at a heating rate of 10 °C/min and at an inflection point of 335 °C.

**Table 2:** Descriptive statistical analysis and significant difference between the studied materials represented in Newtons (N).

	Max	Med	Min	SD	IQ	Mean Rank	Sum of Ranks	Mann–Whitney	Sig. (2-Tailed)
PEEK	3.70	2.248	1.68	0.315	0.443	113.65	10,342.5	2124.5	*p* < 0.001 *
GBP	2.90	2.018	1.43	0.298	0.425	69.35	6310.5		

Max = maximum, Med = median, Min = minimum, SD = standard deviation, IQ = interquartile, * = significant at *p* = 0.05.

**Table 3:** Mean, standard deviation, and significant difference of the deformations (in mm) between PEEK and GBP clasps’ retentive arms and between their reciprocal arms of the studied materials.

	Mean	SD	t-Value	Sig. (2-Tailed)
PEEK (RT)	0.158	0.047	−2.95	0.007 *
GBP (RT)	0.220	0.069		
PEEK (RC)	0.133	0.034	−4.23	<0.001 *
GBP (RC)	0.187	0.0376		

RT = retentive arm, RC = reciprocal arm, SD = standard deviation, * = significant at *p* = 0.05.

**Table 4:** Maximum principal stress values (in MPa) and maximum principal strain values (in mm/mm) of the PEEK and GBP clasps.

	Maximum	Average	Minimum
PEEK (MPS)	9.749	0.1134	−6.395
GBP (MPS)	12.66	0.1612	−6.001
PEEK (MPE)	1.85 × 10^−3^	3.809 × 10^−5^	4.805 × 10^−15^
GBP (MPE)	3.331 × 10^−3^	6.458 × 10^−5^	8.215 × 10^−15^

MPS = maximum principal stress, MPE = maximum principal strain.

## Data Availability

The data presented in this study are available upon reasonable request from the corresponding author.
